# Lifestyle factors and all-cause mortality in long-term cancer survivors: a population-based prospective cohort study

**DOI:** 10.1007/s10654-025-01350-6

**Published:** 2026-01-24

**Authors:** Chunsu Zhu, Melissa S. Y. Thong, Daniela Doege, Lena Koch-Gallenkamp, Heike Bertram, Andrea Eberle, Bernd Holleczek, Alice Nennecke, Annika Waldmann, Sylke Ruth Zeißig, Ron Pritzkuleit, Hermann Brenner, Volker Arndt

**Affiliations:** 1 Unit of Cancer Survivorship, Division of Clinical Epidemiology and Aging Research, German Cancer Research Center (DKFZ), Heidelberg, Germany; 2 Medical Faculty of Heidelberg, University of Heidelberg, Heidelberg, Germany; 3 Cancer Prevention Graduate School, DKFZ, Heidelberg, Germany; 4 Cancer Registry of North Rhine-Westphalia, Bochum, Germany; 5 Bremen Cancer Registry, Leibniz Institute for Prevention Research and Epidemiology - BIPS, Bremen, Germany; 6 Saarland Cancer Registry, Saarbrücken, Germany; 7 Hamburg Cancer Registry, Hamburg, Germany; 8 Institute of Social Medicine and Epidemiology, University of Lübeck, Lübeck, Germany; 9 Cancer Registry of Rhineland-Palatinate, Institute for Digital Health, Mainz, Germany; 10 Institute of Clinical Epidemiology and Biometry (ICE-B), Julius Maximilian University of Würzburg, Würzburg, Germany; 11 Cancer Registry of Schleswig-Holstein, Lübeck, Germany; 12 Network Aging Research (NAR), Heidelberg University, Heidelberg, Germany; 13 Unit of Cancer Survivorship, German Cancer Research Center (DKFZ), Im Neuenheimer Feld 280, 69120 Heidelberg, Germany

**Keywords:** Cancer survivors, Life style, All-cause mortality, Prospective study

## Abstract

The association between healthy lifestyles and mortality in cancer survivors remains inconclusive with few evidence among long-term cancer survivors (LTCS, survived ≥ 5 years post-diagnosis). Our study aims to investigate the association between individual and combined healthy lifestyle factors and mortality in LTCS. We included 6,057 LTCS of breast, colorectal or prostate cancer from a multiple regions study in Germany. A healthy lifestyle score (HLS) comprising alcohol consumption, body mass index (BMI), physical activity and smoking was created and was classified into tertiles with higher tertile indicating healthier lifestyle. We used Cox proportional hazards regression to examine the associations of individual lifestyle factors and HLS with all-cause mortality among LTCS. A total of 2,015 death events occurred over a maximum follow-up period of 12.3 years. Compared with the lowest tertile, participants in the middle and highest tertile experienced a 27% and 32% lower mortality (middle [hazard ratio (HR), 0.73; 95% CI 0.65–0.83]; highest [HR, 0.68, 95% CI 0.61–0.76]). A significant dose-response relationship was observed (p_− trend_ < 0.001). These associations were consistent across different demographic and clinical characteristics. In addition, full adherence to lifestyle recommendations for smoking (HR, 0.51, 95% CI 0.44–0.59), physical activity (HR, 0.78, 95% CI 0.70–0.86) and BMI (HR, 0.87, 95% CI 0.77–0.99) were significantly related to a lower mortality, after full adjustment. Adherence to an overall healthy lifestyle was associated with significantly lower all-cause mortality in LTCS, emphasizing the importance of maintaining and promoting a healthier lifestyle among LTCS.

**Supplementary Information:**

The online version contains supplementary material available at 10.1007/s10654-025-01350-6.

## Introduction

Healthy lifestyle factors (e.g., adequate physical activity and healthy weight) are individually and jointly associated with survival in the general population [1, 2]. However, the relationship between healthy lifestyle factors and better survival in cancer survivors is less clear. Current evidence shows an association between some commonly studied lifestyle factors such as smoking and physical activity and mortality among cancer survivors [3–5]. However, some studies reported that factors such as alcohol consumption had no association with mortality [6]. Moreover, lifestyle factors tend to co-occur and people seldom change a single factor independently from other factors in real life. Focusing on individual lifestyle factors may overlook their cumulative impact on survival outcomes. In our recent systematic review, we summarized existing evidence on the association between combined healthy lifestyle factors and mortality among cancer survivors. We found that survivors reporting an overall healthier lifestyle experienced lower mortality risks [7]. However, most of the included studies assessed lifestyle factors at or shortly after diagnosis, limiting insights of their association among long-term cancer survivors (LTCS, those who have survived ≥ 5 years post-diagnosis). Additionally, the majority of those studies were conducted in the United States limiting external validity. Furthermore, substantial heterogeneity was observed across the studies, which could partially be explained by the difference in the derivation of the healthy lifestyle scores (e.g., diverse components and scoring methods) and study populations (e.g., age, sex, racial composition).

To the best of our knowledge, the evidence on the association between combined lifestyle factors and mortality among LTCS is limited [8]. LTCS represent a heterogeneous population that is relatively understudied and in need of diverse healthcare services [9]. They often experience persistent physiological, psychological, and treatment-related effects that distinguish them from the general populations [10–12]. In addition, studies suggest that LTCS may exhibit different lifestyle patterns compared to the general population. While LTCS are more likely to quit smoking and normalize their BMI, their adherence to dietary recommendations, regular physical activity, and combined healthy behaviors remains suboptimal [13, 14]. Studies on cancer survivors within 5-years post-diagnosis indicated that only approximately 5% adhere to three or more lifestyle recommendations [15, 16], and the adherence level tends to decline over time [17]. These findings suggest that while LTCS may maintain some healthy lifestyles, they are more likely to lag behind the general population in other behaviors, such as physical activity and dietary quality. Moreover, with the combination of an aging population and improvements in cancer screening, diagnosis and treatment, the number of LTCS is growing rapidly [18]. In 2022, there was an estimated 18.1 million cancer survivors in the United States, of whom 70% were LTCS and 48% were very long-term cancer survivors (VLTCS, those who have lived ≥ 10 years post-diagnosis) [19]. In Europe, there were approximately 24 million cancer survivors in 2020, with over 68% and 38% having survived more than 5 years and 10 years, respectively [20]. Similarly, Germany has over 5 million cancer survivors, two-thirds of whom are LTCS [20, 21]. Given the increasing number of LTCS and the potentially high prevalence of unhealthy lifestyle behaviors, having a clearer understanding of the role of an overall healthy lifestyle with respect to prognosis in long-term survivors is a critical public health issue. Therefore, the purpose of this study is two-fold. First, to assess the association of individual and combined lifestyle factors with all-cause mortality among LTCS. Second, to examine whether these associations remain consistent across different characteristics (e.g., age, sex, tumor type) of cancer survivors.

## Methods

### Study design and study population

Data were taken from CAESAR (Cancer survivorship—a multi-regional population-based study), a prospective cohort study of long-term survivors of breast, colorectal and prostate cancer in Germany. A detailed description of this project has been provided elsewhere [22–25]. Briefly, the CAESAR study was initiated in 2008. Survivors diagnosed with invasive breast, colorectal or prostate cancer (ICD-10: C50, C18–C20, C61) between 1994 and 2004 aged 20–75 years old at diagnosis were eligible for participation. Participants were recruited from six cancer registries in Germany (Bremen, Hamburg, North Rhine-Westphalia, Rhineland-Palatinate, Saarland, and Schleswig-Holstein). At initial assessment, individuals who had survived 5 years or more were recruited from August 2009 until April 2011 via postal questionnaires. Of the 14, 526 eligible individuals contacted, 6,057 cancer survivors (42%) completed the full-length questionnaire (breast cancer: 2654; colorectal cancer: 1217; prostate cancer: 2186). Non-participation was associated with a colorectal cancer diagnosis, age at diagnosis under 45 years or over 70 years, and a longer time since diagnosis [26], potentially resulting in a healthier study population. The vital status during follow-up until end of 2021 and date of death were provided by the participating cancer registries.

### Lifestyle behaviors

Four modifiable lifestyle factors were collected through a standardized questionnaire in the CAESAR study, including smoking, alcohol consumption, body mass index (BMI) and physical activity.

### Deriving the healthy lifestyle score

A healthy lifestyle score (HLS) was calculated partially following the World Cancer Research Fund/American Institute for Cancer Research (WCRF/AICR) recommendations on alcohol intake, BMI and physical activity [27, 28]. Alcohol intake was classified as full adherence (never drank), partial adherence (men < 28 g/day, women < 14 g/day), and non-adherence (men > 28 g/day, women > 14 g/day). BMI was calculated from self-reported weight in kilograms divided by height in meters squared, with normal weight (18.5–24.9 kg/m^2^) classified as adherent, overweight (25–29.9 kg/m^2^) as partially adherent, and underweight (< 18.5 kg/m^2^) or obese (≥ 30 kg/m^2^) as non-adherent. Physical activity was grouped by total time spent on moderate- to vigorous physical activity (MVPA) per week: fully adherent (≥ 150 min/week), partially adherent (75–150 min/week) and non-adherent (< 75 min/week).

As smoking was not included in the WCRF/AICR recommendations, the scoring criteria for smoking were based on other common disease prevention guidelines [29]. Current smokers were classified as non-adherent, former smokers were considered as partially adherent, and never smokers were defined as fully adherent.

Each lifestyle factor was assigned 1 point when the recommendation was fully met, 0.5 point when partially met, and 0 point otherwise (Supplementary Table S1), leading to a total score ranging from 0 to 4. The HLS were categorized into tertiles according to the distribution of the sample scores: lowest (0–2), middle (2.5) and highest (3–4).

### Covariates

Considered demographic characteristics include baseline age at survey, sex, self-reported marital status, and self-reported educational level. Clinical data were provided by the cancer registries and included cancer type, cancer stage, and year of cancer diagnosis. Information on cancer treatment was self-reported. Physical comorbidities including stroke, heart attack, heart failure, coronary heart disease, diabetes, osteoporosis, rheumatism, and arthritis were also self-reported.

### Statistical analysis

Baseline characteristics were presented according to the categorical HLS. Continuous variables were described as means and standard deviation (SD) or median and interquartile range (IQR), and categorical factors were presented as absolute values and percentages. Person-years were calculated from the date of answering the initial questionnaire to the date of death, censoring or the end of follow-up, whichever came first.

Cox proportional hazards regression models were used to estimate the hazard ratios (HRs) and 95% confidence intervals (CIs) of lifestyle factors (individual and combined) with all-cause mortality, adjusting for potential confounders. In separate regression models, we first assessed associations for each lifestyle factor (alcohol consumption, BMI, physical activity, and smoking) with all-cause mortality adjusting for demographic characteristics (age at survey, sex, education level, and marital status), clinical factors (tumor type, chemotherapy, radiotherapy, operation, TNM stage, and years since diagnosis), physical comorbidities (stroke, heart failure, heart attack, coronary heart diseases, diabetes, osteoporosis, rheumatism, and arthritis), and the remaining lifestyle factors. Cox proportional hazards regression models were fitted for HLS (in tertiles and continuous) with all-cause mortality, adjusting for demographic characteristics, clinical factors and physical comorbidities. Linear trends were assessed treating HLS tertiles as a continuous variable with Wald tests. The proportional hazards assumption was examined by the Schoenfeld residual method and no violation of the assumption was detected (*p* = 0.325). Subgroup analyses stratified by age, sex, years since diagnosis, cancer type, and presence of cardiometabolic diseases (CMD, one or more of the following diseases including stroke, heart attack, heart failure, coronary heart disease and diabetes) were further conducted. The interaction effect was assessed by adding an interaction term to the fully adjusted model.

To test the robustness of the association between the HLS and mortality, a series of sensitivity analyses were carried out. First, we re-performed our main analyses, excluding each lifestyle factor separately to explore the contribution of each individual lifestyle in the HLS-mortality association. Second, we repeated the analyses in the following scenarios, excluding survivors with: (1) active disease (defined as those who were diagnosed with stage IV or with a self-reported recurrence or metastasis); (2) potentially fatal physical comorbidities at the time of survey, including stroke, heart attack, heart failure; (3) BMI < 18.5 kg/m^2^ as underweight might be a sign of poor health condition; or (4) excluding the first 3 years of follow-up [30]. To assess the potential impact of left-truncation bias, we additionally fitted delayed-entry Cox proportional hazards models using time since diagnosis as the time scale. Missing values were imputed using Monte Carlo Markov Chains multiple imputations (MCMC) based on 25 repetitions. All analyses were conducted in SAS 9.4, and all figures were created with the R language and statistical software (version 4.3.2). Statistical significance was defined by two-sided *p* < 0.05.

## Results

### Baseline characteristics

The mean age (SD) of the LTCS was 69.0 (9.1) years and 3158 (52.1%) were female. Table 1 summarizes the baseline characteristics and imputation results. Participants had survived a median of 8 years at the time of survey, with 80.1% diagnosed 5–10 years previously and 19.9% with ≥ 10 years post-diagnosis. The highest HLS category (3–4 points) included 2,576 participants (42.5%), and 1,409 (23.3%) and 2,072 (34.2%) participants belonged to middle (2.5 points) and lowest (0–2 points) category, respectively. For convenience, we will refer to these categories as tertiles thereafter, despite the different sizes of the highest and middle groups. Participants in the highest tertile were more likely to be female, be higher educated (≥ 12 years), be married or have a permanent partner, and tended to report fewer physical comorbidities.

**Table 1:** Baseline characteristics by the categorical healthy lifestyle score in the CAESAR study

Characteristics	Total (*n* = 6057)		Healthy lifestyle score (0–4)					
			Lowest tertile (0–2)		Middle tertile (2.5)		Highest tertile (3–4)	
	*n* (%)	MI (%)	*n* (%)	MI (%)	*n* (%)	MI (%)	*n* (%)	MI (%)
Age at survey, years, mean (SD)	69.0 (9.1)	69.0 (9.1)	68.2 (9.1)	68.8 (9.1)	69.1 (9.2)	69.8 (8.9)	67.8 (9.0)	68.8 (9.1)
Sex								
Female	3158 (52.1)	52.1	680 (51.0)	51.1	501 (52.2)	51.7	915 (53.6)	54.9
Male	2899 (47.9)	47.9	709 (49.0)	48.9	459 (47.8)	48.3	792 (46.4)	45.1
Education (missing = 120)								
≤ 9	3162 (53.3)	53.0	702 (50.8)	54.3	485 (50.8)	53.9	814 (48.0)	51.4
10–11	1416 (23.9)	24.3	353 (25.5)	24.0	226 (23.7)	23.0	433 (25.6)	25.2
≥ 12	1359 (22.9)	22.7	327 (23.7)	21.7	243 (25.5)	23.1	447 (26.4)	23.4
Marital status (missing = 14)								
Married/permanent partner	4490 (74.3)	74.3	984 (70.9)	70.3	742 (77.4)	75.0	1347 (79.0)	77.1
Others	1553 (25.7)	25.7	403 (29.1)	29.7	217 (22.6)	25.0	359 (21.0)	22.9
Tumor								
Breast cancer	2654 (43.8)	43.8	560 (40.3)	40.7	423 (44.1)	43.1	789 (46.2)	43.1
Colorectal cancer	1217 (20.1)	20.1	323 (23.3)	22.4	177 (19.5)	19.5	314 (18.4)	18.5
Prostate cancer	2186 (36.1)	36.1	506 (36.4)	36.9	360 (37.5)	37.4	604 (35.4)	37.4
TNM stage (missing = 1162)								
I	1446 (29.5)	28.8	321 (28.1)	27.4	225 (29.0)	28.0	444 (31.5)	28.0
II	2266 (46.3)	47.5	524 (45.9)	47.4	357 (46.0)	47.8	648 (46.0)	47.8
III/IV	1183 (24.2)	23.7	297 (26.0)	25.1	195 (25.0)	24.2	316 (22.5)	24.2
Years since diagnosis, median (IQR) (missing = 41)	8 (7–9)	8 (7–9)	8 (6–9)	8 (7–9)	8 (7–9)	8 (7–9)	8 (7–9)	8 (7–9)
Clinical treatments								
Chemotherapy (missing = 484)	2187 (39.2)	39.8	509 (39.3)	39.7	359 (40.5)	39.9	610 (38.3)	39.9
Radiotherapy (missing = 307)	3283 (57.1)	57.1	747 (56.1)	56.4	533 (58.3)	57.2	931 (56.7)	57.2
Operation	4217 (69.6)	69.6	982 (70.7)	68.7	693 (72.2)	69.5	1232 (72.2)	69.5
Physical comorbidities								
Heart failure (missing = 276)	637 (11.0)	11.8	170 (12.7)	14.4	100 (10.7)	11.8	123 (7.5)	9.7
Stroke (missing = 111)	254 (4.3)	4.2	60 (4.4)	5.0	39 (4.1)	4.5	46 (2.7)	3.5
Diabetes (missing = 177)	765 (13.0)	13.2	228 (16.7)	17.5	121 (12.9)	14.1	131 (7.9)	9.2
Coronary heart disease (missing = 206)	715 (12.2)	12.9	185 (13.6)	15.2	115 (12.3)	13.5	144 (8.7)	10.7
Heart attack (missing = 105)	321 (5.4)	5.4	81 (5.9)	6.3	47 (4.9)	5.4	69 (4.1)	4.8
Osteoporosis (missing = 301)	696 (12.1)	12.3	134 (10.0)	10.7	109 (11.8)	13.0	205 (12.5)	13.3
Rheumatism (missing = 337)	422 (7.4)	7.6	115 (8.7)	8.6	69 (7.5)	7.8	95 (5.8)	6.6
Arthritis (missing = 217)	1826 (31.3)	31.7	447 (33.0)	32.8	307 (32.8)	33.0	489 (29.4)	30.2
Lifestyles								
Alcohol consumption (missing = 780)								
0 (male: > 28; female: > 14)	543 (10.3)	12.9	215 (18.1)	24.9	71 (8.3)	10.5	70 (4.4)	4.5
0.5 (male: 0–28; female: 0–14)	2471 (46.8)	40.8	635 (53.4)	44.6	409 (48.0)	43.0	679 (42.6)	36.5
1 (never consume alcohol)	2263 (42.9)	46.3	338 (28.5)	30.4	372 (43.7)	46.5	845 (53.0)	59.0
BMI, kg/m^2^ (missing = 189)								
0 (underweight [< 18.5] or obesity [≥ 30])	1148 (19.5)	20.3	567 (40.8)	43.1	126 (13.1)	12.6	68 (4.0)	6.0
0.5 (overweight [25.0–30])	2579 (44.0)	42.6	608 (43.8)	40.7	521 (54.3)	58.4	633 (37.1)	35.5
1 (normal weight [18.5–25])	2141 (36.5)	37.2	214 (15.4)	16.1	313 (32.6)	29.0	1006 (58.9)	58.5
Physical activity (missing = 1375)								
0 (MVPA:0–75 min/week)	2270 (48.5)	50.1	1161 (83.6)	84.5	544 (56.7)	60.2	257 (15.0)	17.0
0.5 (MVPA:75–150 min/week)	407 (8.7)	6.7	92 (6.6)	5.3	122 (12.7)	9.6	136 (8.0)	6.3
1 (≥ 150 min/week)	2005 (42.8)	43.2	136 (9.8)	10.2	294 (30.6)	30.2	1314 (77.0)	76.7
Smoking (missing = 52)								
0 (current smoker)	642 (10.7)	10.7	346 (24.9)	24.7	56 (5.8)	5.8	35 (2.1)	2.2
0.5 (former smoker)	627 (10.4)	10.4	210 (15.1)	16.3	104 (10.8)	10.8	86 (5.0)	5.3
1 (never smoker)	4736 (78.9)	78.9	833 (60.0)	58.9	800 (83.4)	83.4	1586 (92.9)	92.5

*SD* standard deviation, *IQR* interquartile range, *BMI* body mass index, *MVPA* moderate- to vigorous physical activity, *MI* multiple imputation

### Associations between healthy lifestyles and all-cause mortality

Multivariable associations of the HLS with all-cause mortality among LTCS are presented in Table 2. During a maximum follow-up period of 12.3 years, 2015 all-cause deaths occurred. Each 1-point increase in the HLS was associated with a 22% reduced risk of all-cause mortality in the fully adjusted model (Hazard ratio (HR_adj_), 0.78, 95% CI 0.73–0.83). Compared with the lowest HLS tertile, participants in the middle and highest HLS tertiles had significantly lower all-cause mortality (middle [HR_adj_, 0.73; 95% CI 0.65–0.83]; highest [HR_adj_, 0.68, 95% CI 0.61–0.76]), after adjustment. A significant dose-dependent association was observed (p_− trend_ < 0.001).

**Table 2:** Associations between lifestyle factors and all-cause mortality among long-term cancer survivors

	Deaths/survivors	Crude mortality rate (per 1000 person-years)	Model 1	Model 2	Model 3
			HR (95% CI)	HR (95% CI)	HR (95% CI)
Alcohol consumption					
Nonadherence	245/779	34.5	Ref	Ref	Ref
Partial adherence	771/2471	33.8	0.84 (0.71,0.98)	0.83 (0.71,0.98)	0.90 (0.76,1.05)
Full adherence	999/2807	40.0	0.99 (0.83,1.17)	0.98 (0.83,1.17)	0.99 (0.83,1.17)
BMI					
Nonadherence	496/1228	47.2	Ref	Ref	Ref
Partial adherence	827/2579	34.9	0.72 (0.64,0.80)	0.73 (0.65,0.82)	0.77 (0.68,0.86)
Full adherence	692/2250	33.4	0.79 (0.70,0.89)	0.82 (0.73,0.93)	0.87 (0.77,0.99)
Physical activity					
Nonadherence	1202/3038	45.9	Ref	Ref	Ref
Partial adherence	99/407	25.4	0.74 (0.60,0.91)	0.77 (0.62,0.94)	0.83 (0.67,1.02)
Full adherence	714/2612	28.9	0.71 (0.65,0.79)	0.72 (0.65,0.79)	0.78 (0.70,0.86)
Smoking					
Nonadherence	245/651	42.2	Ref	Ref	Ref
Partial adherence	225/627	40.9	0.73 (0.61,0.88)	0.72 (0.60,0.87)	0.71 (0.59,0.85)
Full adherence	1545/4779	35.4	0.49 (0.43,0.57)	0.49 (0.42,0.56)	0.51 (0.44,0.59)
HLS					
Lowest tertile (0–2)	827/2072	46.2	Ref	Ref	Ref
Middle tertile (2.5)	462/1409	36.1	0.72 (0.64,0.82)	0.72 (0.63,0.81)	0.73 (0.65,0.83)
Highest tertile (3–4)	726/2576	30.1	0.65 (0.58,0.72)	0.66 (0.59,0.73)	0.68 (0.61,0.76)
Trend test			< 0.001	< 0.001	< 0.001
Continuous	2015/6057	36.8	0.75 (0.71,0.80)	0.76 (0.71,0.81)	0.78 (0.73,0.83)

*BMI* body mass index, *HLS* healthy lifestyle score, *HR* hazards ratio, *CI* confidence interval

Model 1 was adjusted for demographic characteristics including age at survey, sex, education level, and marital status

Model 2 was further adjusted for tumor type, chemotherapy, radiotherapy, operation, TNM stage, and years since diagnosis

Model 3 was further adjusted for physical comorbidities (including stroke, heart failure, heart attack, coronary heart diseases, diabetes, osteoporosis, rheumatism, and arthritis), and the other respective individual lifestyle factors. For the HLS models, only physical comorbidities were included

In the analyses of the individual lifestyle factors (Table 2), never smokers (HR_adj_, 0.51, 95% CI 0.44, 0.59) and former smokers (HR_adj_, 0.71, 95% CI 0.59–0.85) had a lower all-cause mortality rate than current smokers. Full adherence to physical activity recommendations was likewise associated with reduced all-cause mortality; partial adherence showed a similar association which though did not reach statistical significance after adjusting for other lifestyle factors. For BMI, lower mortality risks were observed for overweight (HR_adj_, 0.77, 95% CI 0.68–0.86) and normal weight individuals (HR_adj_, 0.87, 95% CI 0.77–0.99) compared to obese or underweight participants. For alcohol consumption, partial adherence to the recommendations initially showed a reduced risk, but this association lost statistical significance after adjusting for other lifestyle factors.

### Subgroup analyses

**Fig. 1:**
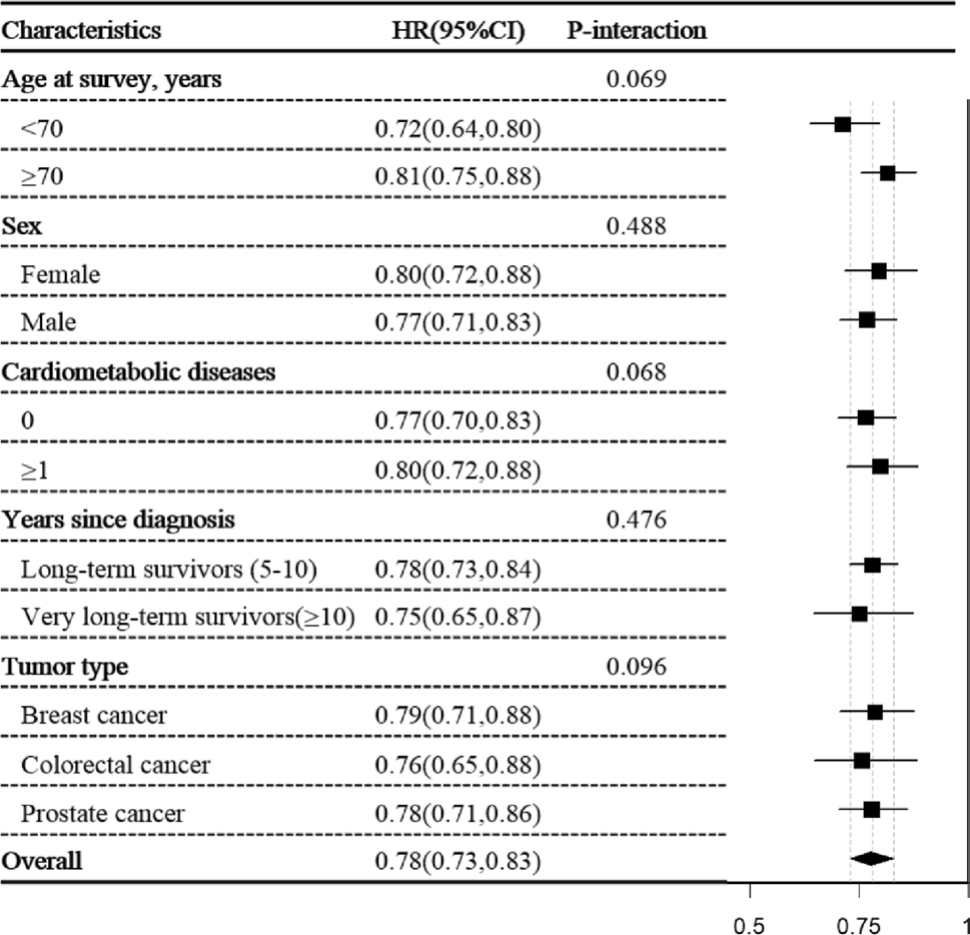
The interaction and subgroup analyses for the association between healthy lifestyle score (continuous) and all-cause mortality. *HR* hazard ratio, *CI* confidence interval. Subgroup specific HRs (95% CIs) were estimated from stratified Cox models; p-interaction values were obtained from models including the interaction term between lifestyle score and the stratifying variable. Models were adjusted for demographic characteristics (including age at survey, sex, education level and marital status), clinical factors (including tumor types, chemotherapy, radiotherapy, operation, TNM stage, and years since diagnosis) and physical comorbidities (including stroke, heart failure, heart attack, coronary heart diseases, diabetes, osteoporosis, rheumatism, and arthritis), as appropriate

The associations of HLS with all-cause mortality appeared to be more pronounced in younger (< 70 years) than in older survivors (≥ 70 years). A per-point HLS increase correlated with a 28% lower mortality in younger survivors (HR_adj_, 0.72, 95% CI 0.64, 0.80)) and 19% in older survivors (HR_adj_, 0.81, 95% CI 0.75, 0.88). However, interaction analyses indicated no significant difference (p_− interaction_= 0.069). These associations remained consistent across subgroups by sex, tumor types, years since diagnosis, and baseline CMD (Fig. 1), and the associations were similar when using HLS tertiles (Supplementary Table S2).

### Sensitivity analyses

**Fig. 2:**
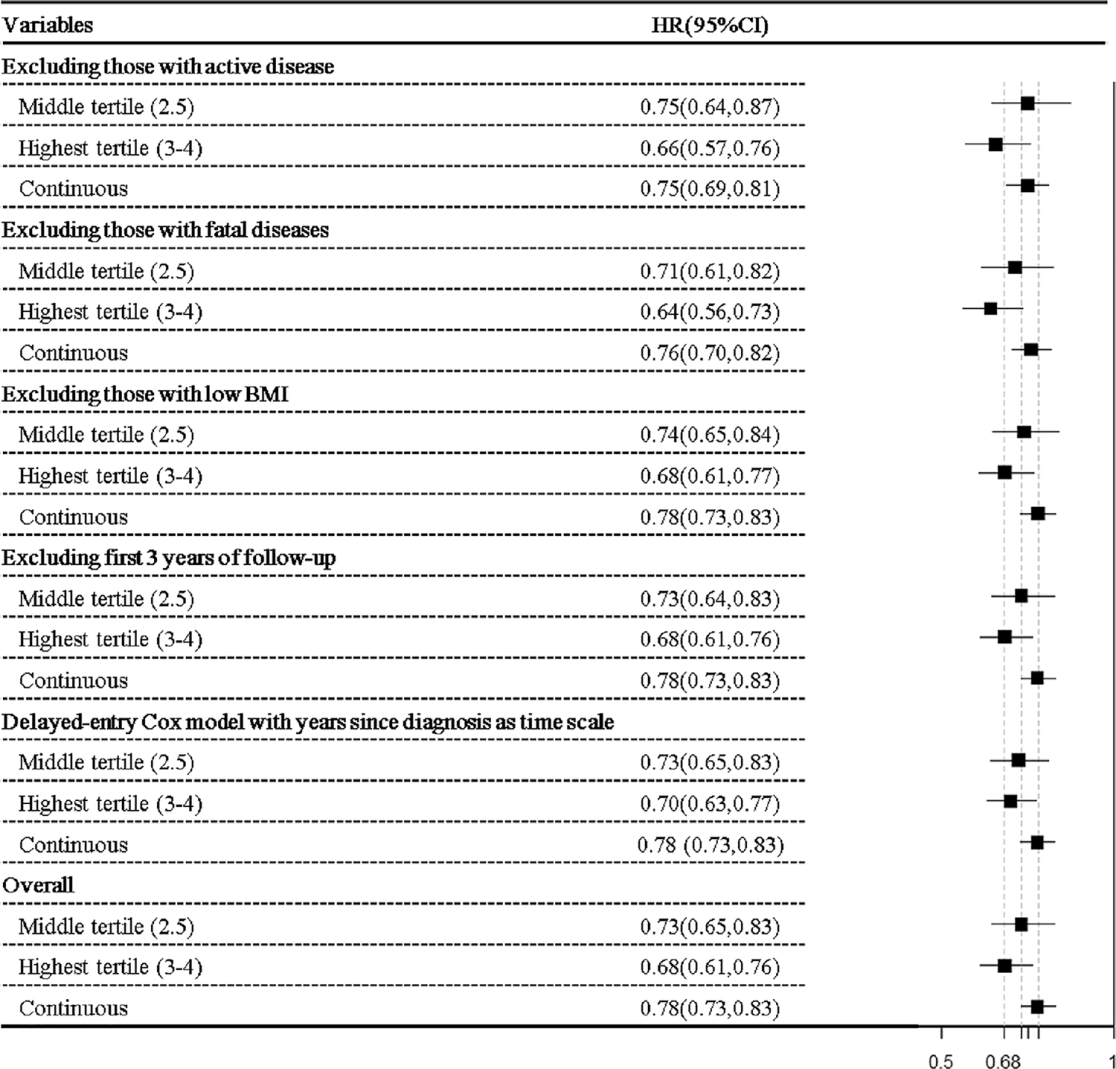
Sensitivity analyses for the association between healthy lifestyle score (categorical and continuous) and all-cause mortality. *HR* hazard ratio, *CI* confidence interval. The lowest tertile of healthy lifestyle score was used as the reference group. Models were adjusted for demographic characteristics (including age at survey, sex, education level and marital status), clinical factors (including chemotherapy, radiotherapy, operation, TNM stage, and years since diagnosis), and physical comorbidities (including stroke, heart failure, heart attack, coronary heart diseases, diabetes, osteoporosis, rheumatism, and arthritis), as appropriate

**Fig. 3:**
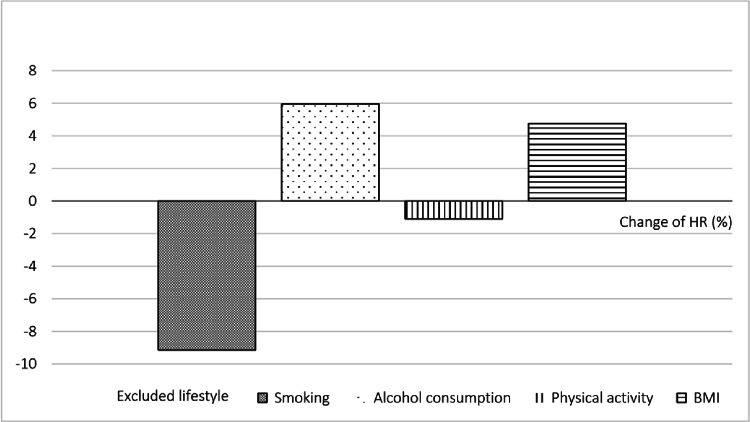
Leave-out analyses depicting the robustness of the healthy lifestyle score by evaluating its sensitivity to the exclusion of each individual lifestyle. Leave-out analyses depict the percent change in hazard ratios (HRs) that represents the relationship between the healthy lifestyle score and all-cause mortality. The downward bars represent a positive percent change in effect, indicating that the association between the healthy lifestyle score and all-cause mortality was attenuated when that lifestyle factor was excluded (the HR increased). The upward bars represent a negative percent change in effect, which indicates the association was strengthened (the HR decreased) when the factor was excluded. Therefore, the lifestyle factor with the largest positive percent increase was the most important contributor to the association of the healthy lifestyle score with all-cause mortality among cancer survivors. BMI: body mass index

Sensitivity analyses showed consistent associations between the HLS and all-cause mortality (Fig. 2). After excluding individuals with active disease, individuals in the middle and highest tertiles had 26% and 38% lower mortality, respectively, than those in the lowest tertile. Similar results were observed after excluding those with potentially fatal physical comorbidities, low BMI, and the first 3 years of follow-up, and in the delayed-entry Cox models. The leave-one-out analyses revealed smoking adherence as the strongest contributor to the HLS-mortality relationship, showing the highest positive percent change in estimate when removed (Fig. 3).

## Discussion

In this prospective study of LTCS from multiple regions in Germany, we found that adherence to a healthy lifestyle behavior, particularly never smoking, engaging in adequate physical activity, and maintaining a healthy BMI, were independently associated with a lower mortality. Adherence to an overall healthy lifestyle (i.e. one-point HLS increase) was associated with 22% lower in all-cause mortality, and these associations remained consistent across age, sex, and years since cancer diagnosis, cancer types, and CMD status.

Although the association between combined healthy lifestyle factors and all-cause mortality has been well established in the general population [31–33], evidence on the role of a healthy lifestyle among cancer survivors, in particular after the fifth year past diagnosis of cancer, has remained inconclusive. LTCS differ from general populations in several aspects, such as higher prevalence of comorbidities and treatment-related sequelae, all of which may influence both lifestyle patterns and mortality risk. In our previous systematic review and meta-analysis on the association between combined HLS and mortality [7], we found that most of the 17 included cohort studies reported a healthy lifestyle to be associated with significantly lower all-cause mortality [34–45], but a few showed no significant association [46, 47]. The pooled results from these studies indicated that cancer survivors adhering to the healthiest lifestyle had a 43% lower risk of all-cause mortality compared to those with the least healthy lifestyle [7]. Our current findings align with this trend, though the observed effect size (32% reduction) is slightly lower. This difference might be attributed to variations in demographic characteristics (e.g., age and sex composition, and geographic region), clinical factors (e.g., cancer types, times since cancer diagnosis) and differences in lifestyle scoring methods (e.g., variations in components and scoring system). For instance, participants in the previous studies were mainly survivors assessed shortly after their cancer diagnosis, when survivors were likely to be undergoing active treatment or experiencing cancer-related symptoms that could substantially modify their lifestyle behaviors. Whereas, our study is the first, to the best of our knowledge, to focus on LTCS who had survived a median of approximately eight years after diagnosis. At this stage, lifestyle behaviors are more likely to have stabilized. In addition, our study is one of the few pertinent studies from outside the United States, thus broadening the evidence base for the potential of HLS with respect to tertiary prevention in cancer survivors.

When investigating the role of individual lifestyle factors, highest level of adherence to recommendations for smoking, physical activity, and BMI were significantly associated with lower in all-cause mortality. Of these factors, never smoking was associated with the greatest risk reduction of 49%. This was further supported in the leave-one-out analyses, which showed that excluding smoking in the HLS resulted in the largest positive percent change in effect size. These findings are in line with previous studies [34, 48], suggesting that smoking cessation remains one of the most impactful lifestyle changes for improving long-term survival in cancer survivors. Interestingly, while overweight and obesity have been reported to be associated with an increased risk of cancer in the general population [49, 50], the HR for the overweight group in our study was lower than that for the normal weight group (0.77 vs. 0.87) when compared with the obese/underweight group. This is not completely unexpected, as an ‘overweight paradox’ has been reported in previous studies [38, 51], suggesting that overweight is associated with improved survival in cancer survivors [6, 48]. While the underlying mechanisms remains unclear, potential explanations for this phenomenon may include greater energy reserve during cancer treatment [52], protective metabolic effects [53], or methodological bias such as reverse causality(e.g., due to tumor-related cachexia) or collider stratification bias [54]. Future investigations should explore whether specific BMI threshold or body composition factors (e.g., fat mass and muscle mass) play a role in the association. Unlike other lifestyle factors, alcohol intake was not significantly associated with all-cause mortality in our study. This finding is in line with other studies. For instance, the Diet, Exercise, Lifestyles, and Cancer Prognosis Study (DELCaP) among breast cancer survivors and a large prospective cohort study in China both found no significant association between alcohol intake and survival [6, 48]. However, the evidence is still inconclusive as previous studies suggested that light alcohol consumption may be a protective factor to mortality in cancer survivors [34]. Given the mixed findings, more evidence is needed to determine the role of alcohol in cancer survivorship outcome.

We further examined whether these associations between the HLS and mortality varied by key clinical and demographic characteristics. Our stratification analyses demonstrated that the associations between a healthy lifestyle and all-cause mortality were largely consistent across different subgroups, including age, sex, time since cancer diagnosis, cancer type, and the presence of CMD. Although the direction was consistent across breast, colorectal, and prostate cancer survivors, slight differences in the magnitude of these associations were observed, which may be explained by cancer-specific characteristics. For instance, breast cancer survivors were often younger at diagnosis and treated with long-term hormone therapy [55]. They may experience distinct metabolic and behavioral effects that influence the lifestyle-mortality relationship. In contrast, colorectal cancer survivors may suffer from persistent gastrointestinal or metabolic issues related to surgery or chemotherapy, while prostate cancer survivors often undergo androgen deprivation therapy, which has been related to adverse cardiometabolic outcomes [56]. These differences highlight the necessity of considering cancer-specific survivorship trajectories when interpreting the effect of healthy lifestyles. Nevertheless, the largely consistent benefit observed across all cancer types suggests that a healthier lifestyle, characterized by never smoking, adequate physical activity, maintaining a healthy BMI and avoiding alcohol consumption, might serve as a broadly applicable recommendation for LTCS. As cancer survivors are now surviving longer, CMD has become the primary comorbidity of concern in this population, even ranking as one of the leading causes of death among cancer survivors [57, 58]. Yet it has remained unclear whether and to what extent the joint healthy lifestyle is associated with all-cause mortality in cancer survivors with CMD. Our findings make a significant contribution to the current evidence, showing that a healthy lifestyle is equally beneficial for LTCS, regardless of CMD status, reinforcing the need to incorporate lifestyle modifications into cancer survivorship care plans.

Our study has several strengths, including a large, well-characterized population of LTCS, the prospective design, and a long follow-up duration. Furthermore, understanding the role of lifestyle factors on LTCS could have significant implications for both clinical practice and public health. Our findings add to the current evidence base by specifically evaluating the impact of lifestyle on mortality among LTCS, an increasingly prevalent but often overlooked group with unique post-treatment risks. Our findings may help health-care providers provide more effective counseling for LTCS, ultimately improving their health outcomes. Notably, over 50% of the LTCS in our study fell into the middle or low healthy lifestyle categories. This finding indicates a significant opportunity for intervention and improvement. Intervention programs should aim to maximize the number of recommended healthy behaviors, with a particular focus on smoking cessation.

However, some limitations should be considered. First, a key limitation is the lack of dietary data in the CAESAR study. Diet is an essential component of lifestyle and has been shown to influence survival among cancer survivor via multiple pathways (e.g., metabolic and inflammatory mechanisms) [59] that are also related to other lifestyle factors included in our score (such as BMI and physical activity). Previous studies have reported that adherence to a healthy dietary pattern, such as Mediterranean diet, is associated with reduced all-cause mortality among cancer survivors [60]. Thus, the omission of diet may lead to an underestimation of the strength of the association between overall healthy lifestyle and mortality. Although a previous study suggested that a HLS with or without diet information yielded similar results for mortality risk [61], the potential contribution of diet remains important and warrants further investigations. Second, self-reported lifestyles at baseline survey cannot reflect pre-diagnosis lifestyles or the changes of lifestyle during follow up. Thus, we cannot tell whether the associations observed are independent of survivors’ pre-diagnosis lifestyle, and whether a change of lifestyles after diagnosis is associated with all-cause mortality. Additionally, cause-specific mortality could not be examined due to the lack of these data in CAESER. Third, although our sample included survivors with the most common cancers among males and females, the low response rate suggest that our results may not be generalizable to all LTCS of these cancer types. Fourth, our analysis focused on LTCS who survived at least 5-years post-diagnosis. This may introduce survival bias, as individuals with unhealthier lifestyles are more likely to have died before being eligible to participate in the survey. Consequently, the here-in described association between lifestyle factors and mortality does not necessarily apply to cancer survivors at an early phase after diagnosis. Finally, to mitigate potential reverse causation, sensitivity analyses were conducted that excluded survivors with active disease, with potentially fatal comorbidities, or with low body weight. These analyses yielded similar results. Despite this, possibility of potential reverse causation and residual confounding by measured or unmeasured factors cannot be ruled out.

In conclusion, adherence to a healthy lifestyle is associated with better survival outcomes in LTCS. These benefits were observed across various demographic and clinical characteristics including age, sex, cancer type, time since cancer diagnosis, and CMD status. Our findings underscore the importance of maintaining or promoting healthy lifestyle behaviors among LTCS. Future research should explore the impact of other lifestyles factors, such as diet, sleep quality and sedentary behaviors, and also lifestyle change on prognosis and quality of life of LTCS.

## Supplementary Information

Below is the link to the electronic supplementary material.

Supplementary Material 1

## Data Availability

Data is available from the authors upon reasonable request.
