# Supplementary material for: Lifestyle factors and all-cause mortality in long-term cancer survivors: a population-based prospective cohort study
Source: Eur J Epidemiol. 2026 Jan 24;41(2):161–71. doi: 10.1007/s10654-025-01350-6 (PMC12975847; doi:10.1007/s10654-025-01350-6)
Supplement: Supplementary file 1 — Supplementary Material 1 [file 10654_2025_1350_MOESM1_ESM.docx]

Table S1. Calculation of healthy lifestyle score

| **HLS** | **Points** | **Adherence level** |
| --- | --- | --- |
| **BMI, kg/m2** |  |  |
| 18.5-25 | 1 | Full adherence |
| 25-30 | 0.5 | Partial adherence |
| <18.5 or ≥30 | 0 | Nonadherence |
| **MVPA (min/week)** |  |  |
| ≥150 | 1 | Full adherence |
| 75-150 | 0.5 | Partial adherence |
| 0-75 | 0 | Nonadherence |
| **Alcohol consumption (g/day)** |  |  |
| 0 | 1 | Full adherence |
| Male: 0-28; Female 0-14 | 0.5 | Partial adherence |
| Male:>28; Female:>14 | 0 | Nonadherence |
| **Smoking** |  |  |
| Never | 1 | Full adherence |
| Former | 0.5 | Partial adherence |
| Current | 0 | Nonadherence |

HLS: healthy lifestyle score; BMI, body mass index; MVPA, moderate- to vigorous physical activity.

Table S2. The Association between HLS tertile and all-cause mortality in different subgroups

| **Characteristics** | **HR (95%CI)** |
| --- | --- |
| **Age at survey, years** |  |
| **<70** |  |
| Middle tertile (2.5) | 0.63(0.48,0.81) |
| Highest tertile (3-4) | 0.55(0.45,0.68) |
| **≥70** |  |
| Middle tertile (2.5) | 0.78(0.68,0.90) |
| Highest tertile (3-4) | 0.75(0.66,0.85) |
| **Sex** |  |
| **Female** |  |
| Middle tertile (2.5) | 0.69(0.56,0.85) |
| Highest tertile (3-4) | 0.70(0.59,0.85) |
| Continuous | 0.80(0.72,0.88) |
| **Male** |  |
| Middle tertile (2.5) | 0.76(0.65,0.89) |
| Highest tertile (3-4) | 0.68(0.58,0.78) |
| **Cardiometabolic diseases** |  |
| **0** |  |
| Middle tertile (2.5) | 0.72(0.61,0.85) |
| Highest tertile (3-4) | 0.65(0.56,0.75) |
| **≥1** |  |
| Middle tertile (2.5) | 0.74(0.61,0.89) |
| Highest tertile (3-4) | 0.75(0.62,0.89) |
| **Years since diagnosis** |  |
| **Long-term survivors (5-10)** |  |
| Middle tertile (2.5) | 0.73(0.63,0.84) |
| Highest tertile (3-4) | 0.70(0.62,0.79) |
| **Very long-term survivors (≥10)** |  |
| Middle tertile (2.5) | 0.76(0.57,1.01) |
| Highest tertile (3-4) | 0.62(0.48,0.80) |
| **Tumor** |  |
| **Breast cancer** |  |
| Middle tertile (2.5) | 0.70(0.56,0.88) |
| Highest tertile (3-4) | 0.68(0.56,0.83) |
| **Colorectal cancer** |  |
| Middle tertile (2.5) | 0.73(0.55,0.97) |
| Highest tertile (3-4) | 0.62(0.48,0.91) |
| **Prostate cancer** |  |
| Middle tertile (2.5) | 0.76(0.64,0.92) |
| Highest tertile (3-4) | 0.72(0.61,0.85) |

HR: hazard ratio, CI: confidence intervals. Healthy lifestyle score (continuous) was used in these models. Models were adjusted for demographic characteristics (including age at survey, sex, education level and marital status), clinical factors (including tumor types, chemotherapy, radiotherapy, operation, TNM stage, and years since diagnosis) and physical comorbidities (including stroke, heart failure, heart attack, coronary heart diseases, diabetes, osteoporosis, rheumatism, and arthritis), as appropriate. The lowest tertile of HLS was used as the reference.
